# The Role of Prosocial Behavior, Aggression, and Assertiveness in Explaining Cyberbullying Victimization Among Youth

**DOI:** 10.3390/ijerph22050760

**Published:** 2025-05-12

**Authors:** Łukasz Nikel

**Affiliations:** Institute of Psychology, University of Zielona Góra, 65-762 Zielona Góra, Poland; l.nikel@wns.uz.zgora.pl

**Keywords:** cyberbullying victimization, aggression, assertive behavior, prosocial behavior, adolescence

## Abstract

This study aimed to examine cyberbullying among youth regarding prosocial behavior, aggression, passive aggression, assertiveness, and passive assertiveness. The study included 183 adolescents (51.4% girls, 39.9% boys, and 8.7% defined as another gender) aged 15 to 18. Aggressive, passive-aggressive, assertive, passive-assertive, prosocial, and cyberbullying behaviors were assessed using self-report questionnaires. The results revealed significant differences in how various responses to conflictual social situations relate to experiences of cyberbullying. Aggressive and passive-assertive behaviors were the strongest predictors of cyberbullying victimization. Furthermore, both behaviors significantly mediated the relationship between prosocial behavior and experiences of cyberbullying. These findings provide insight into practical strategies for dealing with cyberbullying and contribute to our existing understanding of the role of aggression and assertiveness in online harassment.

## 1. Introduction

Nowadays, young people have constant access to mobile devices (e.g., mobile phones, computers, and tablets), providing them with nearly unlimited opportunities to connect with peers and access information [1]. While this accessibility fosters self-development—such as acquiring new knowledge, developing skills, and establishing new connections—it also presents various risks, including cyberbullying [2,3]. Cyberbullying is influenced by multiple psychosocial factors, with one of the most significant being aggression [2,4]. Previous studies on aggression in the context of cyberbullying have primarily focused on the general levels of aggression among perpetrators or victims, e.g., [2,4,5]. A review of recent literature indicates that researchers are increasingly examining specific aspects of aggression, such as reactive and proactive aggression [6,7,8], verbal and physical aggression [2], or instrumental and impulsive aggression [9]. However, most studies in the context of cyberbullying tend to focus on single dimensions of aggression without considering their broader or combined impact on cyberbullying experiences [2,4,5]. Moreover, recent studies suggest that responses to social conflict can be viewed on a continuum [10,11,12], ranging from aggressive to assertive, with intermediate passive behaviors (passive-aggressive vs. passive-assertive behaviors).

Given that few studies have examined different types of aggression in the context of cyberbullying victimization—and none have explored the continuum of behaviors from aggression to assertiveness—this study aimed to investigate the experience of cyberbullying as an example of a social conflict situation through the lens of four distinct types of reactions to conflictual social scenarios rather than relying on the traditional, singular conceptualization of aggression or assertiveness. This approach provides a deeper understanding of which behaviors are most relevant in the context of cyberbullying victimization. Additionally, the study controlled prosocial behavior, as it plays a crucial role in influencing both aggressive and assertive responses.

### 1.1. Cyberbullying

Cyberbullying is a form of bullying that occurs through electronic media [2,13]. It involves repeated acts of aggression intended to cause harm or distress to another person [1,2,13,14]. The prevalence of cyberbullying among young people ranges from 10% to 53%, depending on the population studied and the methods used to measure and define cyberbullying [2,15]. Cyberbullying encompasses various behaviors carried out through digital platforms, including exclusion from online groups, spreading rumors and false information, and making threats, insults, and intrusive messaging [1,15]. While traditional bullying is significantly related to cyberbullying, there are key differences between the two. Compared to traditional bullying, cyberbullying has a broader audience, offers greater anonymity, persists over time, and is more difficult to control [1,13]. Cyberbullying negatively impacts an individual’s psychosocial functioning, regardless of whether they are the perpetrator or the victim [1,15].

#### 1.1.1. Cyberbullying and the Behavioral Continuum: From Aggression to Assertiveness

Aggressive, passive-aggressive, assertive, and passive-assertive behaviors represent four distinct responses to the same social conflict situation. Aggressiveness is characterized by defending one’s rights and needs while violating the rights of others. Assertiveness, on the other hand, involves respecting one’s rights and needs while also respecting those of others [10,16]. Passive aggressiveness and passive assertiveness both involve subordinating one’s own needs and rights; however, the former simultaneously infringes upon the rights of others, whereas the latter does not [10]. These four types of responses can be viewed as forming a continuum. Aggressiveness and assertiveness can be seen as opposing reactions, with their passive forms serving as intermediates. For instance, assertiveness provides a socially acceptable way of managing hostility or anger, whereas aggressiveness does not [10]. Additionally, while aggressiveness and assertiveness are direct responses to social conflict, their passive counterparts represent postponed, indirect ways of reacting to such situations [10,16,17,18].

Research findings indicate significant relationships between aggression, assertiveness, and cyberbullying [2,4,5]. Most studies suggest that aggression increases the risk of involvement in cyberbullying, both as a perpetrator and as a victim [2,4]. However, some studies do not confirm this relationship [19]. In contrast, research on the link between assertiveness and cyberbullying has yielded inconsistent results [5,20]. One study found that assertiveness predicts lower involvement in cyberbullying [20], while another reported the opposite effect [5]. Additionally, some studies found no significant relationship between assertiveness and experiencing cyberbullying [2,5,20], while others identified opposing patterns depending on the population studied—showing a significant positive correlation among Spaniards and a significant negative correlation among Ecuadorians [14].

Some inconsistencies in research findings may be explained by different subtypes of aggression and assertiveness [19]. For example, aggression can be categorized as either reactive or proactive. Reactive aggression, often called “impulsive” aggression, is an immediate response to a perceived or actual threat, such as retaliating against someone. In contrast, proactive aggression, also known as “instrumental” aggression, is goal-directed and not driven by the current situation but instead by an anticipated reward—for instance, discrediting someone for professional gain or social recognition [8,21]. Both perpetrators and victims of cyberbullying report higher levels of both reactive and proactive aggression compared to individuals not involved in cyberbullying [6,22]. Moreover, reactive aggression may be more strongly associated with cyberbullying victimization than proactive aggression, possibly due to impulsive, defensive reactions to frustration [6,7]. On the other hand, among cyberbullying perpetrators, proactive aggression is more strongly linked to cyberbullying than reactive aggression, as it is often used in a more calculated and strategic manner [21].

#### 1.1.2. Cyberbullying and Prosocial Behavior

Prosocial behaviors are actions intended to help another person or a group of people. The key factors motivating prosocial behavior include empathy, altruism, and the principle of reciprocity [23]. These behaviors are associated with skills such as cooperation, helping others, and sharing [5,13]. Additionally, prosocial behaviors serve as a crucial protective factor for children and adolescents, helping to mitigate various challenges, such as externalizing and internalizing disorders [13,24].

The tendency toward prosocial behavior is significantly associated with both aggressive and assertive behaviors, as well as with cyberbullying in both victims and perpetrators [4,5,7,10]. For example, a longitudinal study by Fu et al. (2023) demonstrated the protective role of prosocial behavior in reducing the risk of experiencing cyberbullying [13]. Youth who engage in prosocial behaviors are more liked by their peers and are considered more socially attractive, which decreases their likelihood of being targeted for violence [24,25,26]. Moreover, in environments where antisocial behaviors such as rivalry and competition are prevalent, cyberbullying tends to be more common [5,14]. A study by Zhan et al. (2022) found that social responsibility effectively reduces both perpetration and victimization in cases of violence [27]. However, while prosocial behavior generally protects against cyberbullying, its role in lowering victimization is not always significant [5]. Similar findings have been observed in studies examining the relationship between traditional bullying and empathy [23].

### 1.2. The Present Study

This study aimed to examine the experience of cyberbullying among youth in terms of aggressiveness, assertiveness, passive aggressiveness, and passive assertiveness. Additionally, prosocial behaviors were controlled as they are important determinants of both cyberbullying and aggressive or assertive behaviors. Compared to previous studies that have focused on the reactive/proactive distinction of aggression or examined aggressive and assertive behaviors separately, this study adopted a more detailed conceptualization of aggression while also considering prosocial behaviors—the key factors influencing aggression and assertiveness. This approach provides valuable insights into the causes and conditions underlying behaviors related to the experience of cyberbullying among youth. By framing aggression and assertiveness as a continuum, this study offers a more precise mapping of behaviors associated with cyberbullying. To our knowledge, this is the first study to explore aggressive behaviors along a continuum while also controlling for the variable of prosocial behaviors in the context of cyberbullying. Based on the theoretical framework presented, two hypotheses were proposed.

**Figure 1:**
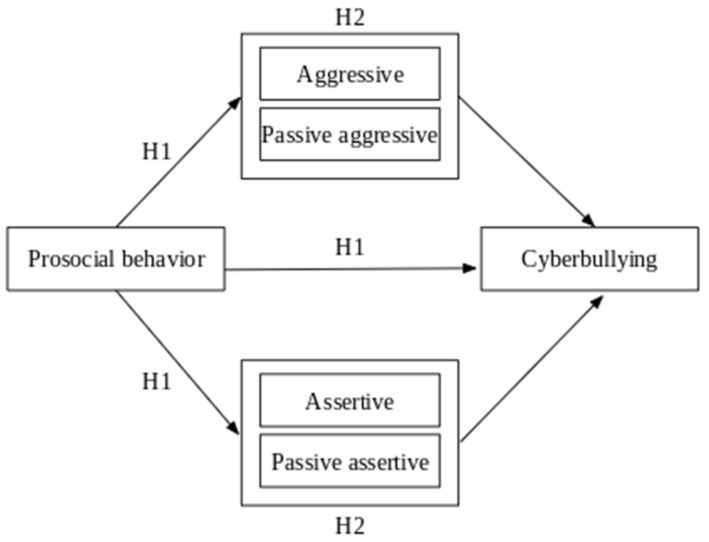
Mediation model of the relationship between prosocial behavior and cyberbullying.

Since prosocial behavior is a key factor in effectively managing social relationships, we hypothesized that it would be negatively related to aggression and the experience of cyberbullying and positively associated with assertive behavior (Hypothesis 1). Furthermore, given that assertive and aggressive behaviors are significantly linked to cyberbullying, we hypothesized that these behaviors would serve as significant mediators in the relationship between prosocial behavior and cyberbullying (Hypothesis 2). The conceptual model is presented in Figure 1.

## 2. Materials and Methods

### 2.1. Participants and Procedure

The study recruited 183 secondary school students from Wrocław, Poland. The eligibility criterion for the purposive sample was the participants’ age. The participants’ ages ranged from 15 to 18 years, with a mean age of 16 (SD = 0.76). The gender distribution included 94 girls (51.4%), 73 boys (39.9%), and 16 participants (8.7%) who selected a different gender option. To perform correlation analysis, a sample size of at least 176 participants is required to detect a medium effect size (r = 0.21, α = 0.05, 1 − β = 0.80), indicating that our sample size was sufficient.

The study was conducted using Google Forms, and participants were not compensated for their participation. School principals, teachers, and parents provided consent for the study to be conducted. Before the study, the participants gave their consent and were informed about the purpose of the research and its confidentiality. The study was registered on the ASPREDICTED website (https://aspredicted.org/ym7uw.pdf) (accessed on 20 February 2025).

### 2.2. Measures

#### 2.2.1. Measurement of Prosocial Behavior

Students completed the prosocial behavior scale from the Big Five Personality Inventory for Children [28] in its Polish adaptation [29]. This scale was chosen because it enabled the study of prosocial behaviors among children and adolescents, which is important given the aims of the current study. A similarly simple format was used in previous related studies, e.g., [10,29,30]. This scale consisted of three items that measure different prosocial behaviors in students. Participants responded using a 4-point scale (definitely not true, not true, accurate, and definitely true) to indicate how much each statement applied to them. An example item is as follows: I try to help others. The estimated internal consistency was *α* = 0.73 and *ω* = 0.75, suggesting that the reliability and internal consistency of the scale were good.

#### 2.2.2. Measuring the Continuum from Aggression to Assertiveness

The youth completed the Aggressive-Assertive Behavior Questionnaire, which assesses four types of reactions (aggressiveness, assertiveness, passive aggressiveness, and passive assertiveness) to a conflictual social situation [31]. This questionnaire allows for the examination of differences between aggression and assertiveness, which is important in relation to the study’s objectives. Instruments of this type demonstrate high construct validity and internal reliability and are widely used in research on aggression and assertiveness among young people, e.g., [10,29]. The questionnaire measures the intensity of the participants’ four reactions. It is based on earlier methods used to examine the continuum of behaviors from aggression to assertiveness, e.g., [17,18] but extends this continuum by including both passive-aggressive and passive-assertive behaviors. The questionnaire consists of four statements depicting a conflictual social situation and serves as a screening tool due to its concise format. Each statement corresponds to one of four possible reactions: aggressive, assertive, passive aggressive, and passive assertive. Participants rate each reaction on a 5-point scale, ranging from 1 (Never) to 5 (Always). This format allows for the calculation of scores for each of the four reaction types (ranging from 4 to 20 points), as well as separate scores for aggression and assertiveness (ranging from 8 to 40 points), and an overall score (ranging from 16 to 80 points), which can indicate social competence. An example statement and possible responses are the following: When someone teases you, I: (a) immediately start teasing them too, (b) leave them alone, I prefer to be friends with someone else, (c) distance myself from the person thinking that I will pay them back anyway, and (d) point out to the person that I do not like what they are doing and ask them to stop. Confirmatory factor analysis confirmed the scale’s structure with four reaction types (aggression, assertiveness, passive aggressiveness, and passive assertiveness): chi-square = 198, df = 98, RMSEA = 0.075, CFI = 0.894, and TLI = 0.870. The model fit values were acceptable [32]. The reliability for the individual scale factors was as follows: aggression *α* = 0.78 and *ω* = 0.78; assertiveness *α* = 0.80 and *ω* = 0.80; passive aggression *α* = 0.64 and *ω* = 0.65; and passive assertiveness *α* = 0.67 and *ω* = 0.68. Given that the scale is designed as a screening tool, the reliability for passive assertiveness and passive aggressiveness is at an acceptable level, while the reliability for aggression and assertiveness is satisfactory [33].

#### 2.2.3. Cyberbullying Victimization

The experience of cyberbullying was assessed using a procedure like those used in the studies by Fu et al. (2023) and Przewłocka (2015) [13,34]. Participants were asked whether they had experienced cyberbullying in the past month. They responded to a single question: How often in the past month did a student or students from your school behave in the following ways towards you: insulted you online or via text message, called you names, expressed hatred towards you, commented on your posts in an offensive manner, or threatened you online? The responses ranged from 1 to 5, with 1 indicating never, 2 indicating at least once a month, 3 indicating several times a month, 4 indicating several times a week, and 5 indicating every day. Higher scores indicated a higher level of cyberbullying.

#### 2.2.4. Plan of Analysis

Analyses were conducted using the Jamovi software (Version 2.6, jamovi, Sydney, Australia) [35,36,37]. In the first step, a simple correlation analysis was performed between the variables assessed in the self-report study to confirm their suitability for the mediation model. Next, a multiple linear regression analysis was conducted to identify the most potent predictors among the four types of behaviors (aggression, assertiveness, passive aggression, passive assertiveness) about the experience of cyberbullying and to confirm the nonlinearity of the variables. A mediation analysis was then performed using the Advanced Mediation Models package [36,37,38] in Jamovi software [35,36,37]. The independent variable was prosocial behavior; the mediators were the types of behaviors that significantly predicted the experience of cyberbullying in the regression model; and the dependent variable was the experience of cyberbullying. Because the skewness and kurtosis indicated a non-normal distribution of the cyberbullying variable, correlations involving this variable were conducted using Spearman’s *rho* coefficient. The mediation analyses were performed using the bootstrapping method (1000 bootstrap samples to estimate the 95% confidence intervals).

## 3. Results

### 3.1. Preliminary Analyses

When analyzing the frequency of adolescents’ responses to the question about their experience of cyberbullying in the past month, the most frequently selected answer was *never* (77.9%, n = 141). The analysis of responses also revealed that 8.3% (n = 15) experienced cyberbullying once a month, 4.4% (n = 8) experienced it several times a month, 3.3% (n = 6) experienced it several times a week, and 6.1% (n = 11) experienced it daily. Two students did not respond to the question about their experience of cyberbullying.

Analyzing the results presented in Table 1, it was found that prosocial behaviors were negatively related to aggression and passive aggression and positively related to assertiveness and passive assertiveness. Moreover, prosocial behaviors were negatively associated with cyberbullying. These results confirm that prosocial behaviors help individuals cope with social situations, including determining how adolescents handle social conflict situations and may serve as a protective factor against cyberbullying. The correlations obtained were consistent with the assumptions of Hypothesis 1. Therefore, regression analysis was conducted in the next step to identify the best predictor of cyberbullying.

### 3.2. Regression Analysis

The tolerance values (1−*R*^2^) and variance inflation factor (VIF) were calculated for the independent variables to check for multicollinearity. It was found that there were no issues with multicollinearity, as the tolerance values were more significant than 0.20 and the VIF was less than 10 [39,40]. Additionally, the Durbin–Watson value was 1.82, indicating no autocorrelation. After confirming that all assumptions were met, regression analysis was conducted with cyberbullying as the dependent variable and the four types of reactions to a conflictual situation as the independent variables (Table 2).

The regression analysis results showed that different types of behaviors in social conflict situations accounted for approximately 14% of the variance in cyberbullying (*F* = 6.94, *p* < 0.001). Analyzing the individual predictors, it was found that aggression was the strongest predictor. The second significant predictor was passive assertiveness, while passive aggression and assertiveness were insignificant. The regression model (Table 2) confirmed that aggression and passive assertiveness explained most of the individual variance in cyberbullying.

### 3.3. Mediation Analyses

**Figure 2:**
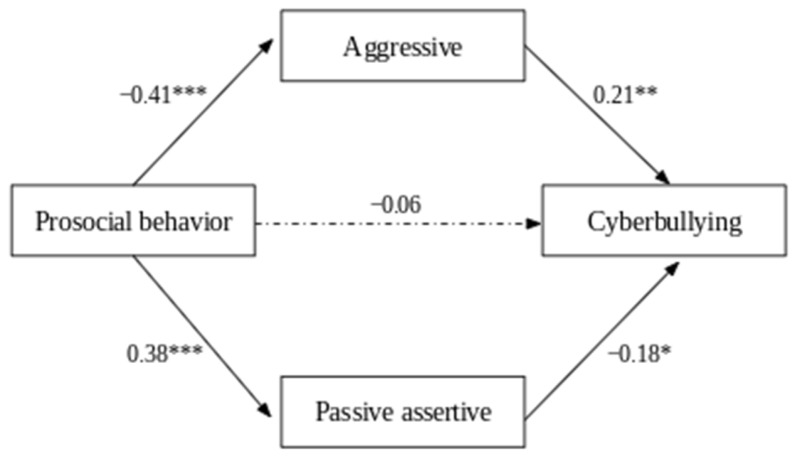
The mediation model with standardized effects of the relationship between prosocial behavior and cyberbullying. The dotted lines indicate nonsignificant relations and the dash line significant relations. * *p* < 0.05, ** *p* < 0.01, *** *p* < 0.001.

Mediation analyses conducted to explain the overall level of cyberbullying (Figure 2) confirmed the good fit of the model to the data, *F* = 9.09, *p* < 0.001, *R*^2^ = 0.13. The results confirmed a significant indirect effect of prosocial behavior through aggression [*b* = −0.05, *boot* 95% *CI* [−0.110, −0.006]] and passive assertiveness [*b* = −0.04, *boot* 95% CI [−0.085, −0.001]] on cyberbullying. These findings support Hypothesis 2. Furthermore, the direct effect of prosocial behavior on cyberbullying was not significant, indicating that prosocial behavior affects cyberbullying indirectly [*b* = −0.03, *boot* 95% *CI* [−0.136, 0.069]]. The total indirect effect of aggression and passive assertiveness as simultaneous mediators was significant, with a value of [*b* = −0.12, *boot* 95% *CI* [−0.228, −0.019]], suggesting that these two variables jointly link prosocial behavior and the experience of cyberbullying. The final mediation model is shown in Figure 2, and a summary of the mediation results is presented in Table 3.

## 4. Discussion

Although the relationship between aggression and cyberbullying has been well documented [4], the relationship between different types of behaviors in social conflict along the continuum from aggression to assertiveness is less clear. To address this gap in research, the present study focused on the effect of aggressive, assertive, passive-aggressive, and passive-assertive behaviors on the experience of cyberbullying. At the same time, prosocial behavior, which is a significant predisposing factor for both aggressive behavior and cyberbullying, was controlled for. Several valuable findings emerged. First, prosocial behavior was significantly associated with all types of reactions to conflictual social situations (positively with assertiveness and passive assertiveness, and negatively with aggression and passive aggression) and negatively with the experience of cyberbullying. Second, the analysis of variables in one model showed that passive assertiveness and aggression are significant mediators of the relationship between prosocial behavior and cyberbullying victimization. These results may serve as valuable references for understanding the ways of coping with cyberbullying experiences and may help explain some inconsistencies found in previous studies.

According to Hypothesis 1, it was expected that prosocial behavior would be negatively associated with aggression and the experience of cyberbullying and positively related to assertive behavior. Our findings confirm this hypothesis: as prosocial behavior decreases, assertive behavior also decreases, while aggressive behavior and the experience of cyberbullying increase. Previous research supports these findings, suggesting that prosocial behavior, which aims to help others, is an important protective factor against various problems in children [24,25,26,41,42,43]. Such behaviors foster a positive socialization cycle [13], which enhances their ability to cope in social situations, shapes positive character traits like self-control, and promotes positive emotions [4,7,10,42,43]. Prosociality encourages the development of strategies based on empathy and cooperation, which is associated with higher assertiveness and lower aggression [4,5,10]. Furthermore, students who engage in prosocial behavior are more liked and attractive to their peers [25,26], and in environments where social responsibility and cooperation dominate, the experience of violence, including cyberbullying, is lower [5,24,27,41].

The current data confirmed Hypothesis 2. More frequent prosocial behaviors in students lead to a greater amount of passive assertiveness and less frequent use of aggression in social conflict situations, which, in turn, contributes to a reduction in the experience of cyberbullying. These results are consistent with previous studies, which have shown that prosocial behaviors are associated with both aggressive and assertive behaviors [4,5,10] and that aggressive and assertive behaviors are linked to the experience of cyberbullying [4,14]. Prosocial attitudes may be a significant motivational factor for behaviors from aggression to assertiveness [10]. This is partly supported by the correlation results in the current study, which show that as behavior progresses along this continuum (aggressive, passive aggressive, passive assertive, assertive), the strength of the relationship decreases and shifts from negative to positive. This suggests that prosocial behaviors play a significant role in predicting behaviors along the aggression-assertiveness spectrum [10]. Moreover, as confirmed by the results of the mediation analysis in this study, students with lower aggression and higher passive assertiveness experience less cyberbullying. These two types of behaviors emerged as the most significant when considering the variance across all four types (aggression, passive aggression, passive assertiveness, and assertiveness). The study’s results indicate that students less prone to aggression and avoiding confrontation are less likely to engage in conflicts and have more excellent peer support, reducing the likelihood of experiencing harassment through mobile devices. These findings align with previous research highlighting the role of emotional and behavioral factors, including prosocial behaviors, in moderating the impact of problematic smartphone use and online engagement in adolescents’ mental health and risk behaviors, such as Internet addiction and peer-related difficulties [42,44]. They are also consistent with evidence showing that deficits in emotional intelligence and empathy among young adults contribute to problematic social media use and its associated negative outcomes [43].

The results obtained in the current study may also help explain some inconsistencies in previous research [5,20]. For example, some studies report a significant negative relationship between assertiveness and the experience of cyberbullying [14], while others found no relationship [2,5]. These inconsistencies may be attributed to subtypes of assertiveness. As shown in the present study, passive assertiveness significantly predicted the experience of cyberbullying, while assertiveness did not when different types of behaviors were controlled in a single model. This suggests that only passive assertiveness may counteract cyberbullying, and assertiveness or confrontation with the perpetrator could, in some cases, even increase the likelihood of experiencing cyberbullying. This is consistent with findings emphasizing that strategies involving confrontation with the perpetrator of violence may exacerbate the experience of cyberbullying [43,45,46]. Moreover, the results may also support a stronger relationship between reactive aggression, rather than proactive aggression, and the experience of cyberbullying [7]. In the analyzed model, as per the definition, passive aggression is postponed and, therefore, more like proactive aggression, whereas aggression is defined as an immediate reaction and, thus, more identical to reactive aggression. The findings from this study indicate the more significant role of aggression than passive aggression in explaining the experience of cyberbullying. This suggests that impulsive, aggressive reactions may escalate retaliation in the form of cyberbullying.

### 4.1. Limitations

This study has several limitations. First, it focused on a sample of Polish adolescents, so the results may be culturally specific and not apply to other cultures. Therefore, it is essential to examine the generalizability of our findings across different cultural contexts. Second, the sample was purposeful, and although it allowed for detecting relationships between the variables studied, it was not representative. Consequently, future studies should aim to replicate the findings with a more representative sample. Third, this was a cross-sectional study, which limits the ability to establish causal relationships. Therefore, longitudinal and experimental studies should be conducted as the next step to determine how prosocial behavior and different types of reactions to conflictual social situations may influence cyberbullying. Fourth, the study variables were measured using questionnaires with data from a single source (i.e., student self-reports). These self-report measures are susceptible to response biases (e.g., recall errors and socially desirable responses), and as a result, the effects may be overestimated due to standard method variance. Future studies should consider combining self-report measures with other data collection methods. Fifth, while prosocial behaviors were examined in this study, other factors may also significantly predict the experience of cyberbullying. Therefore, future research should explore the potential mediating role of various types of reactions to conflictual social situations and additional factors, such as peer relationships. Positive peer relationships may protect against cyberbullying [5,13,44] and promote less aggression and greater assertiveness [10,24,25,41, 43, 47].

### 4.2. Implications

The study makes several significant contributions to the literature. First, it enhances the existing understanding of the role of aggression and assertiveness by explaining the experience of cyberbullying. It was found that aggressive and passive-assertive reactions are the most significant predictors of cyberbullying experiences. Furthermore, an aggressive reaction to a cyberbullying situation may exacerbate the situation, while responding in a passive-aggressive manner may serve as a protective strategy. The results of this study suggest that effective strategies for coping with cyberbullying are those that involve indirect methods, which are passive-assertive actions. These include seeking social support from sources such as parents, helplines, and school professionals, blocking the bully’s account, reporting the incident to administrators, or changing one’s profile on the platform.

In contrast, direct actions such as confrontation or retaliation against the perpetrator are the least effective method [45]. Second, while prosocial behaviors are associated with the experience of cyberbullying, their role is more indirect than direct, primarily influencing behaviors ranging from aggressive to assertive. These findings help explain how developing positive social relationships and improving social competencies can aid in preventing the experience of cyberbullying. This highlights potential intervention goals aimed at reducing the phenomenon of cyberbullying.

## 5. Conclusions

This study tested two hypotheses regarding the relationship between prosocial behavior, aggression, assertiveness, and the experience of cyberbullying. In accordance with Hypothesis 1, it was assumed that prosocial behavior would be significantly negatively related to aggression and the experience of cyberbullying and positively related to assertive behavior. The results obtained confirmed this hypothesis. Students who more frequently engage in prosocial behavior showed less aggression and experienced cyberbullying less frequently while also demonstrating assertive behavior more frequently. Additionally, the mediation analysis confirmed Hypothesis 2, which proposed that assertive and aggressive behaviors could serve as significant mediators in the relationship between prosocial behavior and cyberbullying. Aggressive behaviors and passive assertiveness were found to be significant mediators in this relationship. The findings indicate that aggression positively predisposes students to experiences of cyberbullying, while passive assertiveness negatively predisposes them. Additionally, prosocial behaviors indirectly predict cyberbullying through these two types of behaviors. The results highlight that the relationship between aggression, assertiveness, and the experience of cyberbullying is more complex than previously suggested and points to potential strategies for addressing cyberbullying.

## Figures and Tables

**Table 1:** Descriptive statistics and correlations for study variables.

Variables	1	2	3	4	5	6
1. Cyberbullying	1	−0.16 *	0.24 **	0.23 **	−0.08	−0.27 ***
2. Prosocial behavior		1	−0.41 ***	−0.25 ***	0.34 ***	0.39 ***
3. Aggressive			1	0.73 ***	−0.13	−0.45 ***
4. Passive aggressive				1	−0.04	−0.28 ***
5. Assertive					1	0.28 ***
6. Passive assertive						1
7. M	1.51	9.20	8.07	7.80	11.7	12.8
8. SD	1.13	2.03	3.88	3.42	4.44	3.87

* p < 0.05, ** *p* < 0.01, *** *p* < 0.001.

**Table 2:** Regression analysis predicting cyberbullying on aggressive-assertive continuum.

Model	Cyberbullying		
Predictors	b	SE	β
1. Aggressive	0.07	0.03	0.24 *
2. Passive aggressive	−0.004	0.03	−0.01
3. Assertive	−0.02	0.02	−0.08
4. Passive assertive	−0.05	0.02	−0.17 *

* *p* < 0.05.

**Table 3:** Summary of serial mediation results for cyberbullying symptoms analysis.

Outcome Variables	Predictors	b	SE	β
Aggressive				
	Prosocial behavior	−0.78	0.13	−0.41 ***
Passive assertive				
	Prosocial behavior	0.74	0.13	0.38 ***
Cyberbullying				
	Prosocial behavior	−0.03	0.04	−0.06
	Aggressive	0.06	0.02	0.21 **
	Passive assertive	−0.05	0.02	−0.18 *

* *p* < 0.05. ** *p* < 0.01. *** *p* < 0.001.

## Data Availability

The raw data supporting the conclusions of this article will be made available by the authors upon request.
